# Adverse events in critically ill patients: a cross-sectional study

**DOI:** 10.1590/1980-220X-REEUSP-2021-0481en

**Published:** 2022-05-06

**Authors:** Stefanny Furtado de Assis, Débora Feijó Villas Boas Vieira, Fernanda Raphael Escobar Gimenes de Sousa, Carlos Eduardo de Oliveira Pinheiro, Patrícia Rezende do Prado

**Affiliations:** 1 Universidade Federal do Acre, Rio Branco, AC, Brazil.; 2 Universidade Federal do Rio Grande do Sul, Escola de Enfermagem, Porto Alegre, RS, Brazil.; 3 Universidade de São Paulo, Escola de Enfermagem de Ribeirão Preto, Ribeirão Preto, SP, Brazil.; 4 Universidade Federal do Acre, Programa de Residência Multiprofissional em Terapia Intensiva, Rio Branco, AC, Brazil.

**Keywords:** Nursing Care, Personnel Management, Health Services Administration, Legislation, Nursing, Patient Safety, Intensive Care Units, Atención de Enfermería, Administración de Personal, Administración de los Servicios de Salud, Legislación de Enfermería, Seguridad del Paciente, Unidades de Cuidados Intensivos, Cuidados de Enfermagem, Administração de Recursos Humanos, Administração de Serviços de Saúde, Legislação de Enfermagem, Segurança do Paciente, Unidades de Terapia Intensiva

## Abstract

**Objective:**

To identify the prevalence of adverse events and the critically ill patient’s need for care in an intensive care unit.

**Method:**

This is a cross-sectional study, carried out from January to March 2020. The adverse events investigated were pressure injury, accidental orotracheal extubation, fall, loss of central venous access, and healthcare-associated infection. The number of hours required for patient care was measured by the *Nursing Activities Score*. The categorical independent variables were described by absolute and relative frequencies, and the continuous ones, by central tendency. The magnitude measure was the odds ratio and a confidence interval of 95% was considered.

**Results:**

of the 88 patients evaluated, 52.3% had adverse events, which were associated with a greater need for care, severity, and longer hospital stay. The mean *Nursing Activities Score* was 51.01% (12 h 24 min), with a deficit of 20% to 30% of nursing staff in the unit being identified.

**Conclusion:**

The prevalence of adverse events in the unit is high and the shortage of nursing staff in the unit revealed the need for adequate staffing to reduce the damage caused by the care provided to critically ill patients.

## RESUMO

**Objetivo:**

identificar a prevalência de eventos adversos e a necessidade de cuidado do paciente crítico em uma unidade de terapia intensiva.

**Método:**

estudo transversal, realizado de janeiro a março de 2020. Os eventos adversos investigados foram: lesão por pressão, extubação orotraqueal acidental, queda, perda de acesso venoso central e infecção relacionada à assistência à saúde. O número de horas necessárias para o cuidado do paciente foi mensurado pela *Nursing Activities Score*. As variáveis independentes categóricas foram descritas por frequências absoluta e relativa, e as contínuas, por tendência central. A medida de magnitude foi a razão de chance e considerou-se intervalo de confiança de 95%.

**Resultados:**

dos 88 pacientes avaliados, 52,3% apresentaram eventos adversos, os quais foram associados à maior necessidade de cuidados, gravidade e ao maior tempo de internação. O *Nursing Activities Score* médio foi 51,01% (12 h 24 min), sendo identificado um déficit de 20% a 30% de pessoal de enfermagem na unidade.

**Conclusão:**

a prevalência dos eventos adversos na unidade é alta e o déficit de pessoal de enfermagem na unidade revelou a necessidade de dimensionamento adequado de pessoal para reduzir os danos ocasionados pelos cuidados prestados aos pacientes críticos.

## RESUMEN

**Objetivo:**

identificar la prevalencia de eventos adversos y la necesidad de cuidado del paciente crítico en una unidad de cuidado intensivo (UCI).

**Método:**

estudio transversal, realizado entre enero y marzo de 2020. Los eventos adversos investigados fueron: lesión por presión, extubación oro traqueal accidental, caída, pérdida de acceso venoso central e infección relacionada a la asistencia a la salud. El número de horas necesarias para el cuidado del paciente se midió por la Nursing Activities Score. Las variables independientes categóricas fueron descriptas por frecuencia absoluta y relativa, y las continuas, por tendencia central. La medida de magnitud fue la razón de oportunidad (odds ratio) y se consideró un intervalo de confianza del 95%.

**Resultados:**

de los 88 pacientes evaluados, un 52,3% presentaron eventos adversos, los cuales fueron asociados a necesidad de cuidados más intensa, gravedad y tiempo de ingreso más extenso. El Nursing Activities Score medio fue un 51,01% (12 h 24 min), siendo identificado un déficit entre 20% y 30% de personal de enfermería en la unidad.

**Conclusión:**

la prevalencia de los eventos adversos en la unidad es alta y el déficit de personal de enfermería en la unidad reveló la necesidad de dimensionamiento adecuado de personal para reducir los daños causados por los cuidados prestados a los pacientes críticos.

## INTRODUCTION

Adverse Events (AEs) are unexpected incidents resulting in harm to the patient and are directly associated with the quality of care and/or the lack of care provided. AEs affect, on average, 10% of hospital admissions and reflect the gap between real and ideal care, resulting, in most cases, from the insufficient number of staff to meet the patient’s care needs, especially in the Intensive Care Unit (ICU)^(1–4)^.

Studies have shown that the lower number of nurses per patient and their qualification are directly associated with a higher occurrence of AEs, a higher incidence of burnout, and reduced perception of the quality and safety culture of patient care. In contrast, ICU patients assisted by an adequate number of professionals specialized in intensive care, 24 hours a day, 7 days a week and by nurses with greater autonomy, have longer survival rates and shorter hospital stays. The resources shall be considered by public policies aimed at promoting higher quality care and safety^(2–4)^.

However, in many Brazilian ICUs, the multidisciplinary team works, most of the time, with a number below the one required for critical patients care. This may reflect on missed care, which has been gaining attention in research and is conceptualized as any aspect of patient care that is omitted or delayed, which can have negative consequences, such as increased incidence of adverse events and on patient care safety^(3–4)^. Thus, aiming at safer and higher quality care, it is recommended that nurses use a Patient Classification System (PCS)^(5)^.

The PCS is useful to assess the complexity and hours to be spent for care; however, it is not the most appropriate strategy to assess the need for care in intensive care, as it does not reflect critically ill patients’ needs^(5–10)^. Thus, other tools were suggested to assess the need for ICU care, including the *Nursing Activities Score* (NAS), the *Nine Equivalents Manpower Score* (NEMS), and the *Valoración de Cargas de Trabajo e Tiempos de Enfermería* (VACTE)^(8,10)^. According to research carried out with the three tools, NAS better reflected the care of critically ill patients, when compared to the others^(11)^.

The number of personnel needed to provide safe care to critically ill patients remains a barrier for Nursing professionals, due to the changes made by the Resolution of the Collegiate Board of the National Health Surveillance Agency (RDC/ANVISA nº 26/2012)^(12)^, which changed the number of professional nurses: from 1 nurse for every eight patients, to 1 nurse for every ten patients in intensive care. Such a change may result in increased workload, *burnout,* and higher incidence of AEs^(1–5,12)^.

The increased need for care and the lack of care, assessed by NAS, were associated with the occurrence of AEs in adult ICU patients^(1–4,11)^. Six out of eight studies (75.0%) identified an increase in the occurrence of Pressure Injury (PI), healthcare- associated infection (HAI) and medication error, when the Nursing dimension proposed by NAS was below the patient care needs^(10–11)^. At the moment, NAS is the tool that is closest to the ICU patient’s care needs, as it allows transforming the result of the percentage of time into hours of need for Nursing care to adequately dimension the team with the help of the resolution of the professional class agency^(6–11)^. The greater the adequacy and qualification of Nursing personnel, the greater the likelihood that care will be safer and free from harm^(1–4,11)^.

In the ICU participating in the present study, Nursing dimensioning is carried out through RDC no. 07/2010 and RDC 26/2012^(12)^. The resolution in question presents Minimum Standards for all ICUs in the National Territory. In addition, differences in the complexity of care shall be adequate according to Art. 7, item I, which states that the hospital management shall provide the human resources required for the continuity of care. Moreover, Art. 49 guides the need to measure patient care. In view of this, staffing in each ICU must be carried out according to the assessment of patients’ needs and respecting the minimum number recommended in RDCs 07/2010 and 26/2012^(12)^. It should be noted that the assessment of the need for Nursing care and its relationship with the occurrence of AEs was never evaluated in the ICU of the present study. Thus, the objective of the study is to identify the prevalence of adverse events and the need for patient care through the NAS tool, in a Brazilian ICU.

## METHOD

### Design of Study

Cross-sectional study carried out in an adult ICU of a Brazilian capital in the North region. The study was proposed to answer the following questions: What is the prevalence of adverse events in this ICU? What are critically ill patients’ care needs, in hours, according to NAS tool? Is the number of nurses adequate for the care need identified in the investigated ICU?

### Population and Selection Criteria

The study population consisted of adult patients with at least 48 hours of ICU admission. This length of stay was determined to allow assessment of the patient’s severity and care needs. To assess the need for care, information from the nursing management on the number of nursing staff in the unit was also used.

The ICU of this study has 18 beds to meet the demand from the Brazilian Public Health System (*SUS*) in the capital and region and is part of an Urgent and Emergency Hospital. The main causes of hospitalization are trauma and cardiovascular diseases, including stroke and acute myocardial infarction.

The Nursing team consists of 64 professionals, 12 of which are nurses and 52 are Nursing technicians. This contingent is distributed in morning and afternoon shifts, of six hours each, and a night shift, of 12 hours, with a 60-hour break for rest. All professionals work 30 hours per week. For each shift, there is one nurse and five nursing technicians to assist ten patients, totaling two nurses and nine nursing technicians to care for the 18 patients of this ICU, according to RDC no. 26, of 2012, which amended RDC no. 07^(12)^.

### Data Collection Procedure

Data collection took place daily, in the afternoon, from January 4 to March 17, 2020, in all medical records of hospitalized patients.

The last 24 hours of hospitalization were evaluated by applying NAS daily to all patients and, on the first day of hospitalization, the *Simplified Acute Physiology Score III* (SAPS 3), by a single researcher, through a questionnaire containing the explanatory independent variables and the outcome.

The explanatory independent variables were divided into sociodemographic and clinical ones. The sociodemographic variables analyzed were: sex (male/female) and age (continuous and categorized into < or ≥ 60 years). The clinical variables analyzed were: medical diagnosis (clinical/surgical); length of stay (< or ≥ 7 days); patient severity score, measured by SAPS 3^(13)^ (< or ≥ 50 points) and the patient’s need for care, measured in hours, by NAS^(10)^.

The outcome variables were the AEs recorded in the clinical record (PI, accidental orotracheal extubation, fall, loss of central venous access, and HAI) and discharge/death. The HAIs considered in this study were ventilator-associated pneumonia, urinary tract and bloodstream infections. These AEs were chosen because they are more prevalent in ICUs^(2–4,11)^.

NAS was validated for Brazil by Queijo (dissertation in 2003 and published in 2009^(10)^) and consists of seven major categories, each item has a score and the patient’s score is the sum of the scores of all items according to the direct and indirect nursing care needs. This total represents, as a percentage, how much care time the patient required in 24 hours, with a maximum total of 176.8%. According to the tool’s definition, 100 NAS points are equivalent to 100% of a nurse’s time in the 24 hours^(10)^, where each percentage point equals 14.4 minutes.

SAPS 3 is a tool used to determine the mortality risk of patients at the time of admission to the ICU. It consists of 20 variables divided into three parts: demographic variables/ previous health status (age, comorbidities, previous hospitalization days, origin, and use of vasoactive drugs); diagnostic category (scheduled admission, unscheduled admission, urgency, type of surgery, reason for neurological and cardiological hospitalizations, abdomen and infection) and physiological variables at admission (Glasgow Coma Scale, heart rate, systolic blood pressure, oxygenation, temperature, leukocytes, platelets, pH, creatinine, and bilirubin). Subcategories are scored according to patient severity^(13)^. Theoretically speaking, the lowest value assigned by the score is 16 and the highest is 217 points. A study in the Brazilian population suggested the discriminatory value of SAPS 3 between survivors and non-survivors, around 57–58 points, varying according to the population being studied^(13)^.

The daily records of nurses and physicians were analyzed to calculate the patient’s daily NAS. AEs were confirmed, in the clinical record, by the diagnosis of the unit’s intensive care physician. The admission form was fundamental for the evaluation of SAPS 3.

### Calculation of the Mean Daily Quantitative and Qualitative Factors of ICU Professionals

The sizing of the ICU Nursing team was calculated according to the Resolution of the Federal Nursing Council (COFEN) No. 543/2017, which recommends 52% of nurses, 48% of Nursing technicians, and 18 hours of nursing care for intensive care^(6)^.

The workload was classified according to an adaptation of the workload categories defined by the Epimed Monitor system^®^, in which NAS ≤ 50% is considered light; NAS between 50.1–100%, moderate/high, and NAS ≥ 100%, very high. However, because the mean NAS at the unit is 51.01%, we only chose two categories: NAS ≤ 50%: light; and NAS ≥ 50.1%: moderate/high/very high.

### Data Analysis and Treatment

Categorical variables were described by absolute and relative frequencies and continuous variables by measures of central tendency. The measure of association was the odds ratio (OR), for which the chi-square test was used, or alternatively, in cases of small samples, Fisher’s exact test. A confidence interval of 95% was considered. Data were analyzed using the software SPSS^®^, version 22.0 (SPSS, Chicago, USA).

### Ethical Aspects

This study was approved by the Research Ethics Committee of “Fundação Hospital Estadual do Acre”, by Opinion No. 3.294.722, of April 30, 2019, and ethical principles were observed, in accordance with CONEP Resolution No. 466/2012, of the Council National Health.

## RESULTS

Of the 88 ICU patients studied, 60.2% were clinical (most due to stroke and acute myocardial infarction), 59.1% were male, with a mean age of 49.27 years, mean SAPS 3 of 55.46 points, and NAS of 51.01%. Among the patients, 52.3% had an adverse event, and 39.1% had more than two events. Infection acquired in the ICU (34.1%) was the most frequent adverse event, followed by PI (22.1%). Death occurred in 39.8% of patients (Tables 1 and 2).

**Table 1.** Characteristics of patients (N = 88) in an Intensive Care Unit – Rio Branco, AC, Brazil, 2020.

Variable	n	%
**Sex**		
Male	52	59.1
Female	36	40.9
**Age**		
<60 years	61	69.3
>20 years	27	30.7
Diagnosis		
Clinical	53	60.2
Surgical	35	39.8
Adverse events	46	52.3
**Number of adverse events (N = 46)**		
1 adverse event	28	60.9
2 or more adverse events	18	39.1
Type of adverse event*		
Health care-related infection	30	34.1
Pressure injury	20	22.7
Loss of enteral tube	9	10.2
Loss of central venous access	7	8.0
Accidental orotracheal extubation	2	2.3
Fall	1	1.1
**Length of stay**		
<7 days	41	46.6
≥7 days	47	53.4
**Outcome**		
Discharge	53	60.2
Death	35	39.8
**NAS^†^**		
≤50 points	43	48.9
≥50.1 points	45	51.1
**SAPS 3^‡^**		
<50 points	36	40.9
≥50 points	52	59.1

*Patient could have more than one adverse event; ^†^NAS: *Nursing Activities Score;* ^‡^SAPS 3: *Simplified Acute Physiology Score III.*

**Table 2.** Measures of central tendency, of continuous variables, of patients in an Intensive Care Unit – Rio Branco, AC, Brazil, 2020.

Variable	Minimum	Mean	Maximum	Standard deviation
Age (years)	18.00	49.27	89.00	19.86
Length of stay (days)	2.00	12.82	64.00	13.13
NAS*	27.50	51.01	74.40	9.19
SAPS 3^†^	25.00	55.46	128.0	18.60

*NAS: *Nursing Activities Score; ^†^SAPS 3: Simplified Acute Physiology Score 3*.

AEs were associated with longer hospital stays (>7 days), greater need for care (>NAS), and the greater patient severity (SAPS 3 > 50 points). Length of stay over seven days increased the chance of adverse event by 10.14 times, as did NAS and SAPS 3 over 50 points, which each increased the chance of AEs by three times (Table 3).

**Table 3.** Adverse events in patients in an Intensive Care Unit – Rio Branco, AC, Brazil, 2020.

Variable	Adverse events		*Odds Ratio* (OR)	Confidence interval of 95%
	No	Yes		
**Sex**				
Male	27 (64.3)	25 (54.3)	0.66	0.28–1.55
Female	15 (35.7)	21 (45.7)		
**Age**				
<60 years	28 (66.7)	33 (71.7)	0.78	0.31–1.95
≥60 years	14 (33.3)	13 (28.3)		
**Diagnosis**				
Clinical	21 (50.0)	32 (69.6)	0.43	0.18–1.04
Surgical	21 (50.0)	14 (30.4)		
**Length of stay**				
<7 days	31 (73.8)	10 (21.7)	10.14	3.80–27.08
≥7 days	11 (26.2)	36 (78.3)		
NAS*				
≤50 points	27 (64.3)	16 (34.8)	3.37	1.40–8.10
≥50.1 points	15 (35.7)	30 (65.2)		
**SAPS 3^†^**				
<50 points	24 (57.1)	12 (26.1)	3.77	1.53–9.27
≥50 points	18 (42.9)	34 (73.9)		

*NAS: *Nursing Activities Score; ^†^SAPS 3: Simplified Acute Physiology Score 3*.

The Nursing dimension resulted in 91 nursing professionals for the ICU, with 47 being nurses and 44 nursing technicians, following the stipulated proportion of 52% of nurses and 48% of nursing technicians^(6)^. When considering NAS results, reflecting a scenario that is closer to the unit’s patients’ care needs, it was observed that 81 nursing professionals were required, 42 nurses and 39 nursing technicians. Thus, the deficit of nursing professionals in the ICU is 20.0%, when considering NAS, and 30.0%, when considering the 18 fixed hours of COFEN’s resolution. However, regarding the number of nurses, the difference reaches 71.4% (12/42, referring to the current number of nurses *versus* the number recommended by the unit NAS calculation), as described in Table 4.

**Table 4.** Nursing staff sizing in an Intensive Care Unit – Rio Branco, AC, Brazil, 2020.

Nursing staff sizing	Number of hours of care	Number of beds	Total nursing team professionals	Number of nurses	Nurses (%)	Number of technicians	Technicians (%)	Nursing staff deficit (%)
RDC* No. 26 of 2012 ANVISA^†^ (Current)	12 h 15’	18	64	12	18,9	52	81,1	–
RDC* No. 543 of 2017 COFEN^‡^	18 h	18	91	47	52	44	48	30
NAS**	20 h	2	81	42	52	39	48	20

*RDC: Resolution of the Collegiate Board of Directors; ^†^ANVISA: National Health Surveillance Agency; ^‡^COFEN: Federal Nursing Council; **Nursing Activities Score (NAS).

## DISCUSSION

In the study ICU, 52.3% of patients had an adverse event and 39.1% had more than two events, with PI and HAI being the most prevalent. AEs were associated with longer hospital stays, greater need for care, and greater patients severity. Regarding NAS of the patients in the investigated ICU, a deficit of 20% to 30% of nursing professionals was verified. However, if we consider the care of critically ill patients as exclusive to nurses, this deficit reaches 71.4%.

The frequency of AE was high in the study ICU, similar to a retrospective study carried out in an ICU in Colombia, whose incidence was 52.1%^(14)^; however, the Colombian study used a collection tool “with 16 triggers” to track adverse events, called *Trigger Tool* ^(15)^. In ICUs in Ireland, researchers used a similar tool consisting of 18 triggers and identified an incidence of 12.2%^(16)^. In a study without the use of *Trigger Tools,* in a private and accredited ICU in the city of São Paulo, 8.2% of patients had AEs^(17)^. Such events were associated with greater severity and need for patient care, similar to the present study^(14,17)^.

The use of *Trigger Tools* has been advocated because they can increase the probability of identifying adverse events by ten times when compared to traditional methods of retrospective research in clinical records and reporting systems, which can erroneously guide the direction of improvements in patient safety^(15)^. The frequency of adverse events is multicausal and may vary, besides the research method and data source, according to the characteristics of the patients in the sample, the complexity of the unit, the patient’s safety culture, the length of hospital stay, and the number of professionals and qualification of the health professionals team. The tool shall be included in research on the investigation of adverse events, being a limitation of this study, which we will explain in an appropriate paragraph^(2–4,16,17,18,19,20,21,22)^.

The length of stay can vary from six to 17 days in the ICU^(16–22)^, being longer in public hospitals^(4,17,22)^, similar to the result found in the present study. The longer the length of stay in the ICU, the greater the chance that the patient will have an AE, due to the greater probability of undergoing invasive procedures^(4,17,21,22)^.

Regarding the need for care, a deficit of 20% to 30% of nursing professionals in the unit was identified. This situation is found in most ICUs in Brazil. It should be noted that the deficit is often in relation to the nurse/patient ratio and not in the total number of nursing staff. The insufficient number of nurses to care for patients leads to work overload, increased incidence of burnout, missing care, and of the number of adverse events, mainly in the ICU^(2–4,22)^.

In the ICU studied, there are 12 nurses and 52 nursing technicians. Each shift has two registered nurses and nine nursing technicians for 18 patients, according to RDC n° 26/2012^(12)^. Each technician is responsible for two patients, while each nurse is responsible for nine. However, considering the need for care identified by the application of the NAS in this ICU, nine nurses per shift would be necessary. However, in Brazil, there are three professional categories, including the nursing technician, differing from countries such as the United States and Canada, which have only nurses. However, according to NAS assessment, patients should be cared for by a greater number of nurses, whose adequacy shall be discussed and formalized in the country, aiming at greater safety for critical patients and the reduction of adverse events^(2,5,6,12)^. Furthermore, studies on missing care are suggested, which may explain at least part of the prevalence of AEs^(3,4)^.

The shortage of higher education professionals was also identified in other studies^(17–19)^. ANVISA, through RDC No. 07/2010^(23)^ and No. 26/2012^(12)^, established the minimum criteria for the number of nursing professionals per patient. However, they did not consider the maximum criteria. Thus, managers shall ensure the provision of professionals to meet the critically ill patients’ real care needs^(2,3,10–12)^, according to Article 7 of RDC No. 07/2010^(23)^. In addition, the fact that we live in a continental country with different patient profiles and complexity among ICUs shall be considered^(1,3,4,7,17)^.

Pressure injury was the second most frequent adverse event and this result is similar to previous research carried out in other Brazilian ICUs, whose prevalence ranged from 17.6%^(15)^ to 43.6%^(17)^. In an oncology ICU, PI affected 29.5% of all patients, and most had chronic diseases, diarrhea and enteral nutrition, besides using vasoactive drugs and sedatives for a long time^(24)^. PI may have a higher frequency due to the ease of diagnosis, knowledge, and recording by nurses in the clinical record, unlike accidental extubation, fall, and loss of central venous access and enteral tube^(15,17–24)^.

The loss of the enteral tube and accidental extubation are events that occur, most of the time, during the execution of the bed bath performed by the nursing team^(4,17,18,25)^. Critically ill patients are four times more likely to have enteral tube-related AEs compared to patients receiving minimal care^(5,25)^. Thus, the nursing dimensioning shall be adequate to the care need identified in the unit. Furthermore, the use of an evidence-based care protocol can help reduce these adverse events^(2,3,25)^.

Two cases of accidental extubation were observed in the present study, although none of them were duly notified or recorded in the medical records. It should be noted that both episodes took place during data collection, which indicates that more similar events may have occurred. In a private ICU, there were no records of orotracheal tube loss during the research period^(16)^. This AE increases the workload and the risk of mortality, in addition to extending the patient’s discharge time, which may suggest the need not only for a greater number of professionals, but for greater qualification and training for the care provided^(2–4,26)^.

The patient’s fall from the bed is a difficult AE to be recorded in the ICU^(4,27)^ because, in general, the risk factors are disorientation, frequent urination, ambulatory limitation, absence of a caregiver, postoperative period, and the number of medications administered within 72 hours before the event^(27)^. In addition, continuous surveillance by the healthcare team is a feature of intensive care.

The highest patient severity index, calculated by SAPS 3, was associated with a higher occurrence of AE. Patients with SAPS 3 < 50 points have greater survival rates^(13,28)^ and this is an excellent ICU mortality rate discriminator^(28)^. SAPS 3 mean identified indicates that the patients in this ICU are critically ill on admission, with a high risk of mortality; therefore, they need to be assisted by a greater number of Nursing professionals, particularly those specialized in critical care^(2–4,6,13)^. Studies have shown that critically ill patients assisted by specialists in the ICU, 24 hours a day, seven days a week, by nurses with more autonomy, using protocols and with a lower prevalence of missing care, increases survival and decreases adverse events^(2–4)^.

The frequency of death was high when compared to the frequencies of other ICUs, which ranged from 18.2% to 24.48%^(16,17)^. This result can be explained by the patient’s severity at admission, identified by SAPS 3, longer hospital stay, frequency of adverse events and, probably, the lack of preventive interventions due to the insufficient number of nurses to meet the real need for patient care^(1–4,10–12,29,30)^.

As limitations, the study has a retrospective design and used only information from clinical records, without the use of *Trigger Tool*. In addition, the study was interrupted by the advent of the COVID-19 pandemic, which made it impossible to collect data for 90 days, as previously planned. Also, the nursing staff size was not statistically analyzed with the adverse events variable, not allowing an association to be predicted. However, we cautiously suggest that the number of nurses, according to the scientific evidence explained, may explain the high prevalence of adverse events in the unit studied.

As points to be highlighted, this study identified the high prevalence and factors associated with AEs and suggests that managerial interventions should be carried out aiming at greater safety in patient care at the unit. It is also noteworthy, as an innovative point, that the study allowed the theoretical basis elaboration for the dialogue between the real need for patient care, established by NAS, and the fixed standardization for the number of nurses, guided by RDC No. 26/2012, which does not meet the needs of patients in their different care settings. It is suggested that future research on adverse events should analyze the relationship among the dimensioning, the qualification of professionals, the use of institutional protocols, the presence of multiprofessional visits at the bedside, the use of IHI *Trigger Tools,* and the occurrence of missing care for a broader investigation of adverse events.

## CONCLUSION

The prevalence of adverse events in the unit is high and associated factors included longer hospital stay, greater need for care, and greater patient severity. Furthermore, the deficit of nursing professionals in the investigated ICU is 20.0%, when considering NAS. It is suggested that the number of nurses be revised to meet the real need for care of patients in the unit, aiming at safer nursing care, free from avoidable harm.

## ASSOCIATE EDITOR

Cristina Lavareda Baixinho

## Financial support 

 This paper was supported by the National Council for Scientific and Technological Development (CNPq) in scientific initiation scholarship.
